# A Fast Multi-Scale of Distributed Batch-Learning Growing Neural Gas for Multi-Camera 3D Environmental Map Building

**DOI:** 10.3390/biomimetics9090560

**Published:** 2024-09-16

**Authors:** Chyan Zheng Siow, Azhar Aulia Saputra, Takenori Obo, Naoyuki Kubota

**Affiliations:** Graduate School of Systems Design, Tokyo Metropolitan University, Hino-shi 191-0065, Tokyo, Japan; siow-chyanzheng@ed.tmu.ac.jp (C.Z.S.); aa.saputra@tmu.ac.jp (A.A.S.); t.obo@tmu.ac.jp (T.O.)

**Keywords:** growing neural gas, topological mapping, multiple-camera calibration

## Abstract

Biologically inspired intelligent methods have been applied to various sensing systems in order to extract features from a huge size of raw sensing data. For example, point cloud data can be applied to human activity recognition, multi-person tracking, and suspicious person detection, but a single RGB-D camera is not enough to perform the above tasks. Therefore, this study propose a 3D environmental map-building method integrating point cloud data measured via multiple RGB-D cameras. First, a fast multi-scale of distributed batch-learning growing neural gas (Fast MS-DBL-GNG) is proposed as a topological feature extraction method in order to reduce computational costs because a single RGB-D camera may output 1 million data. Next, random sample consensus (RANSAC) is applied to integrate two sets of point cloud data using topological features. In order to show the effectiveness of the proposed method, Fast MS-DBL-GNG is applied to perform topological mapping from several point cloud data sets measured in different directions with some overlapping areas included in two images. The experimental results show that the proposed method can extract topological features enough to integrate point cloud data sets, and it runs 14 times faster than the previous GNG method with a 23% reduction in the quantization error. Finally, this paper discuss the advantage and disadvantage of the proposed method through numerical comparison with other methods, and explain future works to improve the proposed method.

## 1. Introduction

Recently, the number of elderly people has been increasing in many countries. In Japan, the elderly are projected to account for 38% of the population by 2060 [1]. As a result, the number of elderly people living alone is also increasing, and living alone can make them frail [2]. Therefore, the research on ambient assisted living (AAL) has been conducted rapidly in recent years to provide health and social care services to the elderly through the use of information and communication technology (ICT) in their homes [3]. In fact, intelligent sensing is one of the most important tasks of ICT used in AAL. The basic intelligent sensing tasks are mainly categorized into environmental modeling, environmental monitoring, object detection, and object tracking. Furthermore, long-term monitoring and tracking results are used in behavior pattern analysis and anomaly detection in building environmental maps. Various intelligent sensing systems have been integrated into wearable sensors, smartphones, smartwatches, and wireless monitoring devices with non-intrusive sensors such as image sensors, infrared sensors, and depth sensors to build systems for people monitoring in indoor and outdoor applications [4,5]. The expectation of intelligent sensing in elderly houses and elderly facilities is especially increasing as the price of RGB-D cameras becomes reasonable.

Biologically inspired intelligent methods have been applied to various sensing systems in order to extract features from a huge size of raw sensing data. For example, point cloud data can be applied not only to fall detection [6], human activity recognition [7], and daily life monitoring for elderly people living alone but also to multi-person tracking and suspicious person detection in facilities for the elderly. Basically, a single RGB-D camera is not enough to perform the above tasks in such a situation because as it often encounter occlusion problems, owing to its one-directional and limited view angle [8]. Consequently, multiple RGB-D cameras should be used, but the multi-camera calibration task must be handled [9,10,11]. This is an important task to integrate all camera views into a single world coordinate system [10]. Most studies focus on estimating extrinsic parameters using 3D point cloud data because the intrinsic parameters of recent devices are often available in the device itself. A 3D point cloud is a set of individual points measured in space where each point is represented as its position (x,y,z) in the Cartesian coordinates or the distance (*d*) in 2D arrays. In order to perform multi-camera calibration, we have to detect the overlapping areas in two sets of point cloud data using some features after noise reduction. There are two different approaches to grid mapping and topological mapping for noise reduction and feature extraction. The grid mapping is done in a three-dimensional voxel grid with downsampling [12], but most of the grids are empty, and the search space is still large. Furthermore, the grid size must be determined in advance, and there is still the problem of noise reduction. On the other hand, topological mapping can conduct both noise reduction and feature extraction simultaneously. Therefore, this paper proposes a topological mapping-based 3D environmental map-building method for integrating point cloud datasets measured via multiple RGB-D cameras.

One of the topological mapping methods is growing neural gas (GNG) [13]. GNG is often used to extract and represent a topological structure by using nodes and their edge connectivity in the same dimensional space. We have applied GNG to feature extraction and map building, and we have proposed various types of GNG, such as multi-layer GNG and multi-scale batch learning GNG, in order to improve learning performance. However, we still have two important problems of biased random initialization in the beginning of learning and learning speed in the later part of learning. Since random selection of initial nodes is used, the initial distribution or shape of the topological map may be biased. If the initial topological map is biased, the learning can be computationally expensive. Furthermore, topological mapping based on the large scale of point cloud data requires a great deal of computing time.

Based on the above discussions, in order to reduce the computational time of topological mapping, this paper first propose a fast multi-scale of distributed batch-learning growing neural gas (fast MS-DBL-GNG) by using multiple distributed initial nodes’ selection and a multi-scale distributed batch learning algorithm. Next, random sample consensus (RANSAC) is applied to integrate two sets of point cloud data by using the obtained topological features. In order to show the effectiveness of the proposed method, fast MS-DBL-GNG is applied to perform 3D map building based on topological mapping from several point cloud data sets measured in different directions, with some overlapping area included in two images. The experimental results show that the proposed method can extract topological features enough to integrate point cloud data sets. It runs 14 times faster than the previous GNG methods with a 23% reduction in the quantization error. Due to this better topology extraction, it can achieve a distance error of 0.02 in 3D environment building, which is better than the performance of fast global registration and voxels only.

This paper is organized as follows. Section 2 explains the previous works related to topological mapping, multi-camera calibration, and map building. Section 3 explains the learning process of fast MS-DBL-GNG. Section 4 explains the method of multi-camera 3D environmental map building based on RANSAC using topological features. Section 5 explains the dataset, evaluation metrics, model parameters, and experimental results. Furthermore, this paper compares the experimental results with other methods and discusses the advantages and disadvantages of the proposed method. In Section 6, the paper is summarized and the limitations of this study as well as future works to improve the proposed method are discussed.

## 2. Related Work

### 2.1. Topological Mapping Methods

Originally, topological mapping in mathematics referred to a function between two topological spaces that preserves the properties of the spaces in terms of their topology, but the term topological mapping has been used in artificial intelligence (AI), geology, and robotics, inspired by its original meaning used in mathematics. A topological map used in AI, geology, and robotics is composed of nodes and their edge connectivity like a graph, which is extracted some abstract relationship that exists in real data [14]. This paper only deal with the topological mapping used in AI and robotics.

Various types of topological mapping methods, such as a self-organizing map (SOM) [15], a growing cell structure (GCS) [16], a growing neural gas (GNG), and a self-organizing incremental neural network (SOINN) [17], have been proposed before now. SOM is often used to extract and represent the relationship among high-dimensional data in a low-dimensional data space. GCS and GNG are often used to extract and represent a topological structure by using nodes and their edge connectivity in the same dimensional space. The number of nodes of GCS and GNG is increasing through learning to cover a given data distribution. SOM and GCS can preserve the topological structure, e.g., triangulation, inside one topological map, but SOM and GCS cannot conduct topological clustering. Topological clustering divides a given data set into multiple clusters, while topological mapping is done in each cluster. GNG cannot preserve the same topological structure, but GNG can conduct topological clustering. SOINN was proposed to improve the topological mapping performance of SOM and GNG. The original SOINN consists of two layers of topological networks where the first layer is used to extract topological structures, and the second layer is used to conduct explicit clustering using the typical within-cluster distances and typical between-cluster distances, based on the topological clustering results in the first layer. Table 1 shows the main features and differences of these topological mapping methods.

### 2.2. Growing Neural Gas

The GNG proposed by Fritzke [16] is an incremental network model used for unsupervised learning. It was introduced as an extension of the neural gas (NG) algorithm, which was itself inspired by SOM. GNG differs from SOM and NG in that it can re-adjust the connectivity and delete nodes to form a better topological map. This feature is important because GNG can disconnect two sets of data into two clusters, especially obstacles and traversable paths. However, the limitations of standard GNG are that it converges slowly (in terms of the computation time) and suffers from learning stability issues [18]. To address these limitations, batch learning can be used.

There are various GNG batch learning methods [19,20,21,22]. One method is to use Fuzzy c-means (FCM) to calculate the membership of each network node to each batch data (FCM-BL-GNG) [21]. Doing this can improve the learning stability of standard GNG. However, although FCM-BL-GNG solves the problem of learning stability, it converges slowly because it adds one node to the network in each epoch. Another approach is to accumulate the delta moves of mini-batches of data and then use these delta moves to update network nodes [20]. Doing so ensures stable learning and is faster than using FCM to calculate membership values. These past studies have indeed shown that batch learning performs better than standard GNG in terms of learning stability and speed. However, most works do not fully exploit the use of matrix calculations. Their name implies batch learning, but based on their provided algorithm, batches of data are still input into the network one by one using a for loop. Therefore, in order to perform batch learning without learning data one by one, matrix calculations can be used in the process, as in our previous work (DBL-GNG) [18]. The advantage of using matrix calculations is that learning can be processed in parallel using graphics processing units (GPUs).

To reduce the computation time, Iwasa et al. [22] proposed a multi-scale processing into batch learning to train GNG (MS-BL-GNG). Multi-scale batch processing divides data into multiple small batches of different scales. Their work suggests that not all the data in the input dataset are needed to train GNG in order to obtain topological maps, such as the work of Fernando et al. [20]. To accelerate the network growth, Fernando et al. [19] proposed using an “add-if-silent strategy” method on top of the MS-BL-GNG method to insert nodes into the network in each iteration, and they proposed a dynamic learning structure to reduce data sampling (Fast MS-BL-GNG). The dynamic learning structure is applied to multi-scale batch processing, in which the formal work learns through all multi-scale mini-batches, while the dynamic learning structure learns one mini-batch per scale (per learning phase). This reduces data sampling and speeds up learning. However, this dynamic learning structure requires running full batch learning to converge the network in the final learning phase, which leads to a high computational cost. Moreover, the data distribution of each batch is unbalanced. Therefore, this paper solves the problem of data distribution within each multi-scale mini-batch and applies it to DBL-GNG. This idea comes from the way a forest grows, with each tree having its own soil to grow in. The same goes for mini-batches; each mini-batch should be balanced so that every node in the network can learn in every iteration.

Most previous GNGs have the same problem of their initialization nodes being randomly selected. By having neurons spread throughout a baby’s brain, they are better able to learn and grow, rather than if neurons only appear on one side. Imagine that a faster way to build a forest is to systematically plant trees in different places, rather than randomly choosing a place, because systematic planning ensures that the trees have enough space to grow. Based on this inspiration, GNG initialization must be evenly distributed across the data so that GNG can grow faster. This distribution applies not only to initialization but also to the mini-batches discussed in the previous paragraph. Table 2 shows the main feature differences between variance GNG methods.

**Figure 1:**
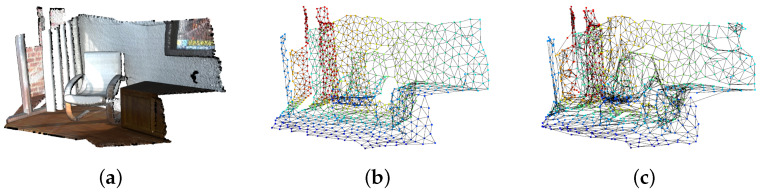
The GNG topological map in the experimental dataset. As can be seen from the figure, when GNG learns a position and color, the connections appear very messy. (**a**) Original point cloud. (**b**) The GNG topology is learned using the position. (**c**) The GNG topology is learned using the position and color.

When learning point cloud data, GNG can learn not only the position of the point but also the color of the point. However, when learning both at the same time, the nodes may be dominated by color features, so the edge connections between the nodes will be messy, as shown in Figure 1. It can be seen that the edge connections in Figure 1c are messier than those in Figure 1b. To address this issue, Toda et al. proposed the different topology GNG (DT-GNG) [23]; where each node connection is depends only on position features.

Based on the above discussion, four key issues that need to be addressed in this study were finally identified. The first is the initialization problem. The initial nodes should be distributed around the point cloud. The second issue is that the multi-scale learning process with a dynamic learning strategy should ignore full batch learning but still achieve convergent learning. The third issue is that the data distribution within each multi-scale batch should be balanced so that each node has data to learn from. The last problem is the edge connectivity problem; the edges should be non-messily so that they can be used for floor path segmentation. For better initialization, a distributed approach with multiple starting points is adopted so that the network can grow faster and more synchronously. To reduce the sampled data, the dynamic learning structure of fast MS-BL-GNG is modified to learn the same data twice in each learning stage so that the network converges in each learning stage and avoids the full batch learning stage. In addition, to get better data distribution in each batch, the data in the initialization stage is used to balance the distribution. Finally, the idea of DT-GNG is adopted, in which each node connection depends only on the position feature. Through these combinations, we named this model fast multi-scale of distributed batch-learning growing neural gas (fast MS-DBL-GNG).

### 2.3. Map Building

**Figure 2:**
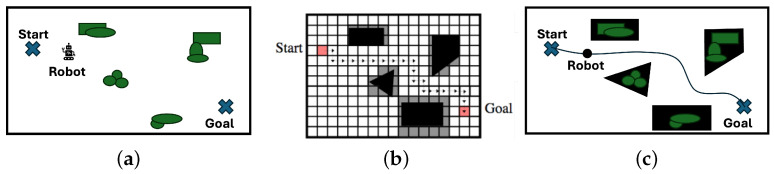
Map building methods for path planning. (**a**) A real environment. (**b**) A grid map. (**c**) A polygonal map.

Map building is one of the most important tasks in robotics. In general, a real environment is approximately represented by a grid map or polygonal map (Figure 2). The grid map is built using the measurement data of a depth sensor. If the smallest size of a grid is larger than the size of the robot, we do not need to take into account the size of the robot for path planning in the grid search space. However, the smallest size should be decided according to the accuracy required for simultaneous localization and mapping (SLAM). On the other hand, a polygonal map is built using approximate polygons. If the polygon is represented by a set of nodes and their edge connectivity, we can use a topological mapping method.

**Figure 3:**
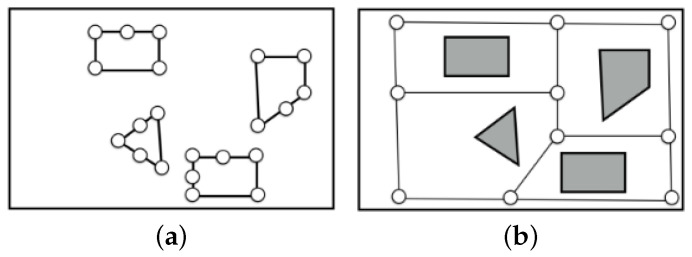
Topological map building. (**a**) Environmental map. (**b**) Roadmap.

**Figure 4:**
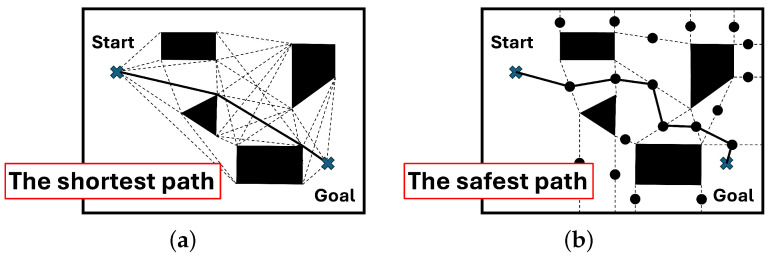
An example of topological path planning in a polygonal map. (**a**) Visibility graph. (**b**) Voronoi diagram.

Topological mapping in robotics means topological map building using a robot. The topological mapping methods are categorized into topological environmental map building and topological road map building (Figure 3). Basically, topological environmental map building is done using the measurement data of depth sensors (external sensor), while topological roadmap building is done using IMU sensors as a result of movement (internal sensors). Topological environmental map building is very useful because we can easily make a visibility graph. In the visibility graph, the shortest path between two points can be generated easily by selecting edges (see Figure 4a). However, it is dangerous for a mobile robot to move along the generated path because the path is adjacent to the vertices of the polygonal objects. To overcome this problem, a Voronoi diagram can be used to generate a safe path far away from any vertices (see Figure 4b). The best path can be generated using a visibility graph and a Voronoi diagram, according to a multi-objective function predefined for path planning. Furthermore, this path planning can be considered one of the topological map building methods. In this way, the applicability of topological environmental map building is very high.

Next, the relationship between SLAM and multi-camera calibration will be discussed. In the SLAM, a robot often uses the local features of a grid map to estimate the current position and posture of the robot from the measurement data using the following equation:

(1) $$y_{R}\leftarrow f\left(x_{R},W\right)$$

where $y_{R}$ is the current position and posture of the robot, $x_{R}$ is a measurement data set from the robot, and *W* is the current map. Meanwhile, the robot builds and updates the current map by using the current position and posture according to the following equation:

(2) $$W\leftarrow g(x_{R},y_{R})$$

In this way, the map is updated sequentially as the robot moves. These two equations are nested within each other. Therefore, if the localization error is large, the corresponding map is corrupted, and if the current map is corrupted, the localization error is also large. The basic idea of SLAM is related to 3D grid mapping for multi-camera calibration. The multi-camera calibration can be formulate as follows:

(3) $$y_{C2}\leftarrow f\left(y_{C1},x_{C1},x_{C2}\right)$$

where $y_{C1}$ and $y_{C2}$ are the extrinsic parameters of cameras 1 and 2, respectively, and $x_{C1}$ and $x_{C2}$ are the measurement data of cameras 1 and 2, respectively. In this equation, $y_{C1}$ is given in the global coordinate system, or $y_{C1}$ is estimated from the relative coordinate system. If measurement data include much noise, it is difficult to solve the multi-camera calibration. Therefore, noise reduction method and/or feature extraction method are needed. The above Equation (3) can be reformulate as follows:

(4) $$y_{C2}\leftarrow f\left(y_{C1},W_{C1},W_{C2}\right)$$

where $W_{C1}$ and $W_{C2}$ are the map using the features extracted from the measurement data of the cameras 1 and 2, respectively. We need to use noise reduction in a grid map because the relationship between measurement data is not used in the grid map building, but we do not need to use noise reduction because the noise is automatically removed through topological learning. The multi-camera calibration is discussed in detail in the next subsection.

### 2.4. Multi-Camera Calibration

To achieve 3D-environment mapping in a room, expertise is required to calibrate the cameras so that they merge and match each camera point cloud into the same world-coordinate system. One traditional method is to use a calibration object to establish a reference for the camera in order to accurately measure distances and angles. Common calibration objects include chessboard planes [24,25,26,27], spherical objects [9,28], or cube objects [10]. Instead of using calibration objects as reference objects, some studies have been conducted using human skeletal joints [11,29]. However, when using the camera in a home and office environment, the camera may be slightly rotated due to certain events (such as cleaning), and the user may need to spend time calibrating it and may not know how to re-perform the calibration [3]. Therefore, there is a need for automatic calibration between multiple cameras without using any calibration objects or human skeleton joints, which may sometimes fail to accurately estimate human skeleton joints.

The registration method is a common method to find the transformation relationship between two point clouds [10,26,30]. The registration method can be divided into local registration and global registration. A well-known local registration method is to match several corresponding points in a point cloud using the iterative closest point (ICP) registration technique [31]. ICP has been the mainstay of geometric registration in research and industry for many years. The input is two point clouds and an initial transformation that roughly aligns the source point cloud with the target point cloud, with Color-ICP providing more accurate and robust registration along the tangent plane than previous point cloud registration algorithms [32]. However, ICP is suitable for fine-tuning matching, and it is not suitable for global matching purposes when two point clouds are located at different locations (very different initializations). To solve this problem, several corresponding points can be manually selected in the two point clouds for ICP registration, but manual operation is still required. To do this automatically, global registration can be used, which does not require the two point clouds to be close enough to each other. A common method in global registration is to use the random sampling consensus (RANSAC) algorithm for global registration [33]. This technique repeatedly generates hypotheses by randomly sampling three or more correspondences, and it evaluates the quality of the generated hypotheses based on the spatial consistency of the correspondences. However, RANSAC takes time to perform registration because it involves iterative sampling, so another, faster technique is fast global registration (FGR) [34]. FGR does not involve iterative sampling, model fitting, or local refinement, and it can quickly optimize the line-process weights for a small number of correspondences.

Point clouds can be used to calibrate multiple cameras, but a single RGB-D camera may output 1 million data points, which is a huge processing cost for the multi-camera calibration process. Voxel filtering methods can be used to downsample the point cloud in order to reduce processing costs [12]. But if the point cloud is downsampled too much, the details of the point cloud may be lost, and corresponding points cannot be found to match the two point clouds. At the same time, the downsampled point cloud does contain some noise values [35], which may cause false matches. To reduce the noise, GNG can be used to obtain important points from the point cloud. In addition, GNG can also be used to generate a topological map in order to reduce the processing cost of matching [13]. As discussed in Equation (4), a map is used, which is a topological map generated via GNG as follows:

(5) $$W_{C1},W_{C2}\leftarrow h\left(x_{C1}\right),h\left(x_{C2}\right)$$

where h(·) is the topological map extraction function.

## 3. Fast MS-DBL-GNG

**Figure 5:**
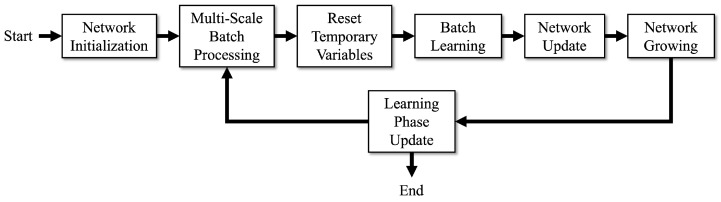
The overall process of fast MS-DBL-GNG. The network is first initialized by creating multiple starting points in the point cloud. Then, based on the initialization, the point cloud data are rearranged and split into multi-scale mini-batches. For each mini-batch, it learns twice. During the learning process, it first resets the temporary variables and then learns the mini-batch in a batch matrix calculation manner. After learning is completed, the temporary variables are used to update the network node weights and edges. Then, it calculates the total number of nodes that should be added and, next, adds them to the network. The process is repeated until all multi-scale mini-batches are gone through.

This section explains how to convert point clouds into topological maps using the proposed method, fast MS-DBL-GNG. Fast MS-DBL-GNG has five main tasks, namely network initialization, multi-scale batch processing, batch learning, network update, and network growth. The overall process of fast MS-DBL-GNG is shown in Figure 5. The main notations used in fast MS-DBL-GNG are shown in Table 3.

### 3.1. Network Initialization

**Figure 6:**
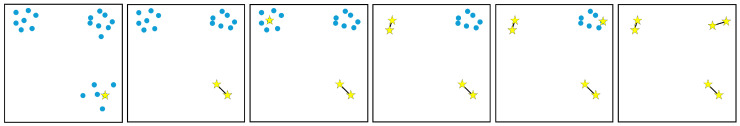
An example of distributed initialization for three starting points. The circles are data, and the asterisks are nodes. First, a node is randomly selected in the last batch of data as the first starting point. Then, the third closest node is selected and connected. After that, the first *B* data surrounding it are deleted. The next starting point is selected in the area farthest from the current starting point. The same process is repeated until all three points are initialized.

In standard GNG, the initialization randomly inputs two data into the network as nodes. This initialization is slow, and it takes time to converge the network. Therefore, we intended to use distributed initialization. Distributed initialization places multiple starting points evenly on the point cloud. First, a starting point is randomly selected. Then, the next starting point is generated farther away from the current point, and then the same process is repeated without returning to the previously set starting point area so that all starting points can be evenly distributed across the data. Figure 6 shows an example of the distributed initialization process for three starting points.

First, the total number of starting points, η, must be defined, and the batch size, *B*, is calculated as follows (this batch size is different from the multi-scale batch size in the next section):

(6) $$B\leftarrow \frac{D}{\eta }$$

Next, randomly select a data node from a subset of the data, in which the subset is the batch at the end of the data (when the index data, *i*, is greater than a certain threshold), as follows:

(7) $$w_{m}\in_{R}\left\{x_{i}|i>D-B\right\},\forall i\in X$$

where $\in_{R}$ is randomly selected data from the subset, X is the complete dataset, and $w_{m}$ is the *m*-th node inserted into the network, *W*. The first *m* is 1. Then, calculate the distance, *d*, from the current node to all data, and then sort the data in order of distance from nearest to far, as follows:

(8) $$d\leftarrow \sum X^{2}-2\left(w_{m}\cdot X^{T}\right)$$

(9) $$X\leftarrow x_{i},\forall i\in \arg\;\mathrm{sort}\left(d\right)$$

where argsort(d) return the indices of distance in ascending order. The distance formula is modified Euclidean distance because we only need to know the data in the sorted index. Next, select the third closest node as the neighbor node of the current node, and connect them with an edge, as shown below:

(10) $$w_{m+1}\leftarrow x_{3}$$

(11) $$c_{k,j}\leftarrow 1,\forall k\in \left\{m,m+1\right\},\forall j\in \left\{m+1,m\right\}$$

where *c* is the adjacency matrix of the network. This study choose the third closest node so that it has a different growth direction. If it is too close, then two nodes may grow in the same direction. Next, before remove the most recent batch from X, store it in $\hat{X}$ for later multi-scale tasks, as follows:

(12) $${\hat{X}}_{t}\leftarrow \left\{x_{i}|i\leq B\right\},\forall i\in X$$

where ${\hat{X}}_{t}$ is the *t*-th batch explored. Then, remove the nearest batch from the X as follows:

(13) $$X\leftarrow \{x_{i}|i>B\},\forall i\in X$$

Finally, repeat Equations (7)–(13) η times. The distributed node initialization process is shown in Algorithm 1.
**Algorithm 1** Network Initialization.**Input:** X,η▹ Data and the total number of starting points, respectively.**Output:** W,c▹ Nodes and edges, respectively. m←1 B← Equation (6)▹ Define the batch size. **for** 
t←1,…,η 
**do**  $w_{m}\leftarrow$ Equation (7)▹ Randomly select data from the end batch.  d← Equation (8)▹ Calculate the distance between the selected node and all data.  X← Equation (9)▹ Sort data based on distance.  $w_{m+1}\leftarrow$ Equation (10)▹ Select the third nearest data as a neighbor node.  c← Equation (11)▹ Creates an edge connecting the selected node to the neighbor node.  ${\hat{X}}_{t}\leftarrow$ Equation (12)▹ Save the closest-distance batches from the data.  X← Equation (13)▹ Remove the closest-distance batches from the data.  m←m+2▹ Update the number of network nodes. **end for**

### 3.2. Multi-Scale Batch Processing

**Figure 7:**
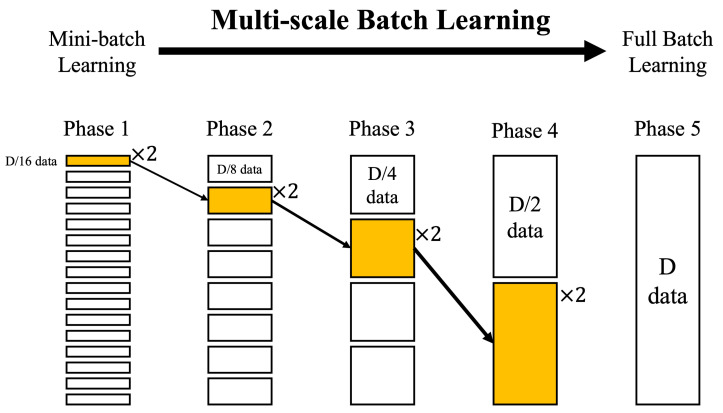
The fast multi-scale batch-learning process. Data are learned from a small scale (**left**) to a full batch (**right**). However, this study avoid learning the full batch and instead learn the same mini-batch twice in each learning phase.

After the network is initialized, the next step is to learn the entire batch of data. Full batch learning is computationally expensive. Therefore, to speed up learning, this study split the data into small batches, just like the learning technique in deep learning. However, only learning mini-batches of the same data scale cannot ultimately obtain a refined topological network. Therefore, this study adopts multi-scale batch processing and divides the learning into multiple learning phases. Each learning phase learns mini-batches of different scales, from a small scale to a large scale. Therefore, more nodes are added at the beginning, and finally, the topological map is fine-tuned to match the point cloud. To speed up learning and make it converge faster, this study avoid learning with a full batch and instead learn the same mini-batch twice in each learning phase. The fast multi-scale batch-learning process is shown in Figure 7.

**Figure 8:**
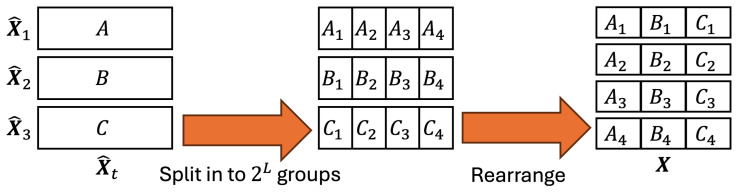
The example procedure for balancing the data distribution in each mini-batch, where η is 3, and *L* is 2. First, divide each set of data ${\hat{X}}_{t}$ into $2^{L}$ groups and then rearrange the data to data X.

Before batching, the data must be rearranged to achieve the same data distribution in each mini-batch. First, shuffle the data arrangement of each ${\hat{X}}_{t}$. Next, divide each ${\hat{X}}_{t}$ into $2^{L}$ groups, and then rearrange each group into data X for the following multi-scale batch processing. The schematic diagram of this process is shown in Figure 8. By doing this, we can ensure that the data in the following mini-batches are distributed.

The average of the losses over the mini-batch can be used to update the weights of the network. As shown in Figure 7, where *D* is the number of data points of X, multi-scale learning divides the data into *L* learning phases, and each learning phase increases the mini-batch size until the full batch is reached. The formula for obtaining the mini-batch data size is as follows:

(14) $$\lambda^{l}\leftarrow \frac{D}{2^{\left(L-l\right)}},\forall l\in \left[1,\cdots ,L\right]$$

where $\lambda^{l}$ is the batch size of the *l*-th learning phase. After obtaining the batch size of each learning phase, use it to split the data points into a small mini-batch. In learning phase 1, use the first mini-batch for training, and in subsequent learning phases, use the second mini-batch to avoid repeated data in learning phase 1. The formula for obtaining mini-batch data is given below:

(15) $$\begin{array}{c} X^{l}\leftarrow \left\{x_{i}|\tau <i\leq \tau +\lambda^{l}\right\},\forall i\in X\\ \tau \leftarrow \lambda^{l}\times \min\left(l-1,1\right) \end{array}$$

where τ is the starting anchor point for forming a mini-batch, and $X^{l}$ is the mini-batch data for the *l*-th learning phase. The work of Fernando et al. [19] increases the learning phase when the number of network nodes reaches the predefined number of nodes in the learning phase. However, this paper increase the learning phase when learning the same mini-batch twice. The multi-scale batch processing process is shown in Algorithm 2.
**Algorithm 2** Multi-scale batch processing.**Input:** $\hat{X},\eta ,L$▹ η initialization data, the total number of starting points, and the total number of learning phases, respectively.**Output:** W,c▹ Nodes and edges, respectively. **for** 
t←1,…,η 
**do**  ${\hat{X}}_{t}\leftarrow$ shuffle $\left({\hat{X}}_{t}\right)$▹ Shuffle all initialization data. **end for** $g\leftarrow B/2^{L}$▹ Define the group size. **for** $r\leftarrow 1,\ldots ,2^{L}$ **do**▹ For each group.  **for** t←1,…,η **do**▹ For each initialization data.   **for** i←1,…,g **do**    $X_{r\times B+t\times g+i}\leftarrow {\hat{X}}_{t,g\times r+i}$▹ Rearrange the data.   **end for**  **end for** **end for** $\lambda^{l}\leftarrow$ Equation (14) **for** l←1,…,L **do**▹ For each learning phase.  $\lambda^{l}\leftarrow$ Equation (14) ▹ Batch size of each learning phase.  $X^{l}\leftarrow$ Equation (15)▹ Data of each learning phase.  **for** 1,…,2 **do**▹ Learn each mini-batch twice.   Reset temporary variables   Batch learning   Network update   Network growing  **end for** **end for**

### 3.3. Batch Learning

For GNG learning in each mini-batch, first reset the temporary variables of the batch. The temporary variables of the batch are delta movements, $\Delta W^{*}$, node activations, $A^{*}$, and temporary edges, $\tilde{c}$. The next step is to find the first and second winner nodes for all data in the mini-batch. Based on the idea of different topologies [23], this study only calculate the distance of position features. The formula is as follows:

(16) $$d_{i,k}\leftarrow \sqrt{\sum {\left(X^{l,Pos}\right)}^{2}-2\left(X^{l,Pos}\cdot {W^{Pos}}^{T}\right)+\sum {W^{Pos}}^{2}}$$

(17) $$s_{1_{i}}\leftarrow \underset{\forall i\in d}{arg min}\left(d_{i}\right)$$

(18) $$s_{2_{i}}\leftarrow \underset{\forall i\in d\wedge i\neq s_{1_{i}}}{arg min}\left(d_{i}\right)$$

where $d_{i,k}$ is the distance matrix between the *i*-th data and the *k*-th node, and arg min(·) is the return index of the minimum value in the list. Pos is the position features. As can be seen from the equation, there is no for loop involved, but all distances from all data to each node can be obtained. Although arg min(·) looks like a for loop searching for the smallest index, in some specific libraries (such as the NumPy library), hardware-specific tricks are used to speed up vectorized operations on the CPU. Therefore, using arg min(·) is faster than a for loop. Here, we have done a little trick. Given the features of $X^{l}$ and *W*, the first three features are position features (Pos), and the last three features are color features (Col). This makes it easier to perform matrix calculations.

Next, use the identity matrix $I_{m}$ to calculate the error for each node. The equation is calculated as follows:

(19) $$E\leftarrow E+\left(\sum_{i=1}^{\lambda^{l}}\left({\left(I_{m}\right)}_{s_{1}}\times d\right)\right)\times \alpha$$

where *E* is the cumulative error of the node, α is the learning rate of the first winner node, ${\left(I_{m}\right)}_{s_{1}}$ returns the node matrix of $s_{1}$ as $\left[\lambda^{l}\times m\right]$, and *m* is the total number of nodes in the network. The default value of α is 0.5.

Next, accumulate the delta movement instead of updating the network immediately. This update uses position features and color features. The delta movement is a [m×F] matrix. The calculation is different from the previous equation because it involves the number of features, *F*, of the node. The equation is as follows:

(20) $$\Delta W^{1}\leftarrow \Delta W^{1}+\left({\left(I_{m}\right)}_{s_{1}}^{T}X^{l}-{\left(W^{T}\times \sum_{i=1}^{\lambda^{l}}{\left(I_{m}\right)}_{s_{1}}\right)}^{T}\right)\times \alpha$$

where $\Delta W^{1}$ is the delta matrix based on winner nodes $s_{1}$.

Afterwards, the neighbor nodes of the winner nodes $s_{1}$ need to be updated. This equation is the same as the last equation, but it uses an adjacency matrix. The equation is derived as follows:

(21) $$\Delta W^{2}\leftarrow \Delta W^{2}+\left(c_{s_{1}}^{T}X^{l}-{\left(W^{T}\times \sum_{i=1}^{\lambda^{l}}c_{s_{1}}\right)}^{T}\right)\times \beta$$

where β is the learning rate of neighbor nodes, $c_{s_{1}}$ returns the connected node matrix of $s_{1}$ as a $\left[\lambda^{l}\times m\right]$ matrix, and $\Delta W^{2}$ is the delta matrix based on neighbor nodes of $s_{1}$. The default value of β is 0.01.

To average the delta movement, count the number of times a node is selected for an update in each batch. Count the number based on the winner node, $s_{1}$, and the neighbor nodes of $s_{1}$, as follows:

(22) $$A^{1}\leftarrow A^{1}+\sum_{i=1}^{\lambda^{l}}{\left(I_{m}\right)}_{s_{1}}$$

(23) $$A^{2}\leftarrow A^{2}+\sum_{i=1}^{\lambda^{l}}c_{s_{1}}$$

where $A^{1}$ is the activation count of each node based on $s_{1}$, and $A^{2}$ is the activation count of each node based on neighbor nodes of $s_{1}$.

After that, the next step is to create a temporary edge connecting the first and second winner nodes, as follows:

(24) $${\hat{c}}_{k,j}\leftarrow 1,\forall k\in \left\{s_{1},s_{2}\right\},\forall j\in \left\{s_{2},s_{1}\right\}$$

Fast MS-DBL-GNG do not use the edge age in batch learning. The reason is that batch learning performs all node updates in one batch; therefore, edge age is not important since the connection of $s_{1}$ and $s_{2}$ is continuously updated in every learning batch.

Equations (16)–(24) are the learning process in batch learning, just like obtaining the loss value in deep learning. The process of batch learning is shown in Algorithm 3.
**Algorithm 3** Batch learning.**Input:** $X^{l},\Delta W^{1},\Delta W^{2},A^{1},A^{2},\hat{c}$▹ They are a batch of data, delta movements, node activation counts, and temporary edges.**Output:** $\Delta W^{1},\Delta W^{2},A^{1},A^{2},\hat{c}$ d← Equation (16)▹ Calculate the distance of each data to each node. $s_{1}\leftarrow$ Equation (17)▹ Get the winner node for each data. $s_{2}\leftarrow$ Equation (18)▹ Get the second winner node for each data. E← Equation (19)▹ Update the error node of $s_{1}$. $\Delta W^{1}\leftarrow$ Equation (20)▹ Update the delta movement based on $s_{1}$. $\Delta W^{2}\leftarrow$ Equation (21)▹ Update the delta movement based on the connected nodes of $s_{1}$. $A^{1}\leftarrow$ Equation (22)▹ Update the node activation count based on $s_{1}$. $A^{2}\leftarrow$ Equation (23)▹ Update the node activation count based on the connected nodes of $s_{1}$. $\hat{c}\leftarrow$ Equation (24)▹ Update the edge strength based on $s_{1}$ and $s_{2}$.

### 3.4. Network Update

Next, use the obtained values to update the network. First update the node features, as follows:

(25) $$W\leftarrow W+{\left({\left(\Delta W^{1}\right)}^{T}\times \frac{1}{A^{1}+\epsilon }\right)}^{T}+{\left({\left(\Delta W^{2}\right)}^{T}\times \frac{1}{A^{2}+\epsilon }\right)}^{T}$$

where ϵ is the epsilon value to prevent a zero division error, which is set to $1\times 10^{-4}$. Then update the network edges as follows:

(26) $$c_{k,j}\leftarrow \left\{\begin{array}{cc} 1, & {\hat{c}}_{k,j}>0\\ 0, & {\hat{c}}_{k,j}=0 \end{array}\right.,\forall k,j\in \hat{c}$$

Next, delete those isolated nodes and the edges connected to them. The index of the isolated node is obtained as follows:

(27) $$\chi^{Isolated}\leftarrow \left\{k|\sum_{j=1}^{N(w_{k})}\left(c_{k,j}\right)=0\right\},\forall k\in W$$

where $\chi^{Isolated}$ stores the indexes of the isolated nodes, and N(·) returns the neighbor nodes. Last, the cumulative error across all nodes is reduced using the discount factor δ as follows:

(28) $$E_{k}\leftarrow E_{k}\times \delta ,\forall k\in W$$

where the default value of δ is 0.5.

Since some nodes are not updated in a batch of learning, this study proposes to remove those inactive nodes as follows:

(29) $$\chi^{Inactive}\leftarrow \left\{k|A_{k}^{1}=0\right\},\forall k\in A^{1}$$

where $\chi^{Inactive}$ stores the indexes of the inactive nodes. However, this deletion of inactive nodes cannot be done frequently because some nodes may be active in the next learning batch. Therefore, set a probability of 10% to perform inactive node deletion. The process of network update is shown in Algorithm 4.
**Algorithm 4** Network update.**Input:** $\Delta W^{1},\Delta W^{2},A^{1},A^{2},\hat{c}$▹ They are delta movements, node activation counts, and temporary edges.**Output:** W,c,E▹ They are the network nodes, the adjacency matrix, and node errors. W← Equation (25)▹ Update network nodes based on delta movement. c← Equation (26)▹ Update network edges based on temporary edges. $\chi^{Isolated}\leftarrow$ Equation (27)▹ Get those isolated node indexes. $W,c,E,A^{1}\leftarrow$ Remove($\chi^{Isolated}$)▹ Delete those related variables based on node indexes. $m\leftarrow m-|\chi^{Isolated}|$▹ Update the number of network nodes. E← Equation (28)▹ Perform error discounting on all nodes. **if** U([0,1]) > 0.9 **then**▹ Gives a 10% chance to perform the following action.  $\chi^{Inactive}\leftarrow$ Equation (29)▹ Get those inactive node indexes.  W,c,E← Remove($\chi^{Inactive}$)▹ Delete those related variables based on node indexes.  $m\leftarrow m-|\chi^{Inactive}|$▹ Update the number of network nodes. **end if**

### 3.5. Network Growing

After the network nodes are updated, the next task is to insert a new node between the highest-error node and its highest-error neighbor node. To speed up the growth, first calculate how many extra nodes should be added for the current batch learning using the percentile function as follows:

(30) $$g\leftarrow \sum E>P_{E}\left(p\right)$$

where *g* is the number of nodes that should be grown in the current batch, *P* is the percentile function, and *p* is the filter probability value. This study set *p* to 0.85 by default. By doing this, it ensure that network growth is distributed.

First, get the maximum error node, $q_{1}$, and its maximum error neighbor node, $q_{2}$, and then insert a new node, $q_{3}$, between them, as shown below:

(31) $$q_{1}\leftarrow \underset{\forall k\in W}{arg max}\left(E_{k}\right)$$

(32) $$q_{2}\leftarrow \underset{\forall k\in N(w_{q_{1}})}{arg max}\left(E_{k}\right)$$

(33) $$w_{q_{3}}\leftarrow 0.5\times \left(w_{q_{1}}+w_{q_{2}}\right)$$

Then, delete the edge between $q_{1}$ and $q_{2}$. At the same time, create two edges connecting $q_{3}$ to $q_{1}$ and $q_{2}$, respectively, as follows:

(34) $$\begin{array}{cc} c_{k,j} & \leftarrow 0,\forall k\in \left\{q_{1},q_{2}\right\},\forall j\in \left\{q_{2},q_{1}\right\}\\ c_{k,j} & \leftarrow 1,\forall k\in \left\{q_{1},q_{2},q_{3},q_{3}\right\},\forall j\in \left\{q_{3},q_{3},q_{2},q_{1}\right\} \end{array}$$

Then reduce the errors of nodes $q_{1}$ and $q_{2}$ by a ratio, ρ, and assign the $q_{1}$ and $q_{2}$ node errors to the $q_{3}$ node error as follows:

(35) $$\begin{array}{cc} E_{q_{1}} & \leftarrow E_{q_{1}}\times \rho \\ E_{q_{2}} & \leftarrow E_{q_{2}}\times \rho \\ E_{q_{3}} & \leftarrow (E_{q_{1}}+E_{q_{2}})\times 0.5 \end{array}$$

where the ratio ρ is set to 0.5. To prevent overfitting, set the maximum number of nodes, *M*, to 2000. The process of network growth is shown in Algorithm 5.
**Algorithm 5** Network growing.g← Equation (30)▹ The total number of nodes to grow.**for** 1,…,g **do** **if** m < M **then**▹ The network does not exceed the maximum number of nodes.  $q_{1}\leftarrow$ Equation (31)▹ Get the maximum-error node.  $q_{2}\leftarrow$ Equation (32)▹ Get the maximum-error neighbor node of $q_{1}$.  $w_{q_{3}}\leftarrow$ Equation (33)▹ Create a new node in the network.  c← Equation (34)▹ Create an edge between $w_{q_{1}}$ and $w_{q_{2}}$.  E← Equation (35) ▹ Update the node errors of $w_{q_{1}}$, $w_{q_{2}}$, and $w_{q_{3}}$.  m←m+1▹ Update the number of network nodes. **end if****end for**

## 4. Multi-Camera 3D Environmental Map Building

**Figure 9:**
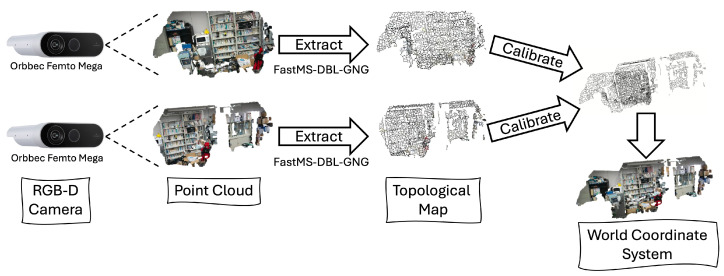
The overall system architecture for automatic calibration using topological mapping. First, set up two Orbbec cameras in the environment to observe two different and partially overlapping areas. Then, extract RGB point clouds based on the intrinsic parameters, depth, and RGB color provided via the cameras. Use the proposed method, fast MS-DBL-GNG, to extract topological maps from each point cloud. These topological maps are then used to extract histogram features, followed by calibration using RANSAC and Color-ICP. Through calibration, extrinsic parameters are obtained and used to calibrate the point cloud to the world coordinate system.

This section introduces an explanation of how to use the topological maps extracted from fast MS-DBL-GNG for multi-camera 3D environmental map-building tasks. An overview of the entire architecture system is shown in Figure 9. First, each RGB-D camera captures a point cloud. Then, the proposed method, fast MS-DBL-GNG, is used to extract the topological map of each point cloud. These topological maps are then used for calibration, and the obtained extrinsic parameters are then used to calibrate the camera point cloud view to the world coordinate system. Furthermore, can use the obtained extrinsic camera parameters for other fine-tuning tasks. For example, they can be used to obtain better 3D human skeleton models from different viewpoints, as shown in our previous work [36].

### 4.1. Topological Mapping-Based Multi-Camera Calibration

A point cloud may contain more than 1 million data. Therefore, to reduce the amount of data, voxel downsampling can be performed. Voxel downsampling creates a uniformly downsampled point cloud from the input point cloud using a regular voxel grid. This technique is traditionally used in the field of computer graphics to subdivide the input space and reduce memory costs but still represent the overall shape and structure of the original data [35]. It consists of two steps. The first step is to divide the points into voxels, and the second step is to average all the points within each occupied voxel. Choosing the size of the voxel grid can be challenging, and voxels that are too large may oversimplify the data, resulting in the loss of fine details [12]. To avoid the loss of details, this study set the voxel size to 0.01.

Subsequently, the downsampled point cloud is input into fast MS-DBL-GNG to output the topological map. Since the topological map (network node weights) contains 3D positions, we can use this information for normal estimation. First perform a k-dimensional tree (K-D Tree) using the topological graph to perform space partitioning. This helps speed up the process of finding the closest node. For each node, its 30 closest nodes are used to calculate the normal of that node. These nodes are used to compute the covariance matrix. Then, a robust algorithm [37] is used to obtain the eigenvalues and eigenvectors for estimating the normal vector.

After the normal of each node is obtained, use it to compute fast point feature histograms (FPFHs) [38]. FPFHs describe the local geometry around a point in a 3D point cloud. Use the FPFH method [38] to create features for each node in the topological map based on the estimated normal and its position. The 50 nearest nodes are used to create the features. However, these output features do not contain color information, so this study concatenate the color of the node to the output feature.

The features of each node in one topological map (point cloud A) are used to match it with another topological map (point cloud B). First, use RANSAC for global registration [39], picking three random nodes from a topological map (source) and matching them to another topological map (target) using their node features. Two strategies are set when pruning wrong pairwise alignments; the first one is to check whether the aligned point cloud is smaller than 0.01, and the second one is to check whether the length of the line formed by two nodes is greater than a 0.9 similarity. This study set the maximum number of iterations to 0.1 million. After the transformation from RANSAC is obtained, apply its transformation to the source topological map and then perform local refinement using colored point cloud registration (Color-ICP) [32]. This study set the maximum iteration to 100.

### 4.2. 3D Environmental Map Building Strategy

**Figure 10:**
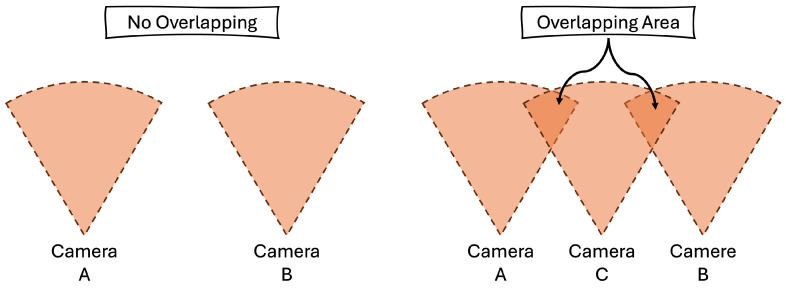
The challenge of calibrating three or more point clouds is that the two selected point clouds do not have any overlapping areas. In addition, there is no camera arrangement ID between these cameras.

Based on previous studies [11,29], two point clouds are used for calibration, provided that there is some overlap between the two point clouds. One of the challenges of calibrating three or more point clouds to build a 3D environment map is that the overlapping area of the point clouds may change, and the arrangement of the accessed point clouds is not fixed, as shown in Figure 10, where two camera views do not have any overlapping area, and a third camera is needed to partially overlap them. In addition, there is no camera arrangement ID between these cameras.

**Figure 11:**
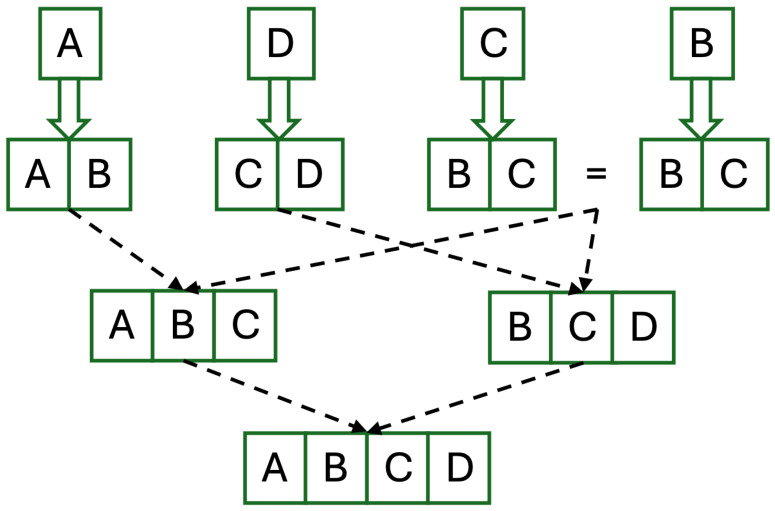
Each point cloud is first merged with the best matching point cloud. Duplicate merges are removed. And then, the matching is performed again until all point clouds have been used.

To solve this problem, we can set one point cloud as the main point cloud and compare the remaining point clouds with it. Then, merge the best matching point cloud with the main point cloud as the latest main point cloud, and then perform the next round of matching again until all point clouds are merged. However, matching a small point cloud with a large one can be problematic because the large point cloud will contain more noisy data and lead to false matches. Based on this problem, this study proposes the expansion of the overlapping area of each point cloud by merging it with the best matching point cloud. Each enlarged point cloud then tries to match with other enlarged point clouds, and then the best matches are merged, and the same process is repeated until all point clouds are used. The best match is determined by the fitness result of RANSAC. Figure 11 illustrates the concept of this idea. In this study, a topological map was used for such repeat matching. First, extract the topological map of each point cloud using fast MS-DBL-GNG. Then, perform RANSAC on each topological map with other topological maps. Each topological map was then merged with the topological map with the highest fitness value in RANSAC. Last, perform Color-ICP to fine-tune them. If there were identical merged point clouds, only kept one. Repeat the matching and merging process again until all topological maps were connected.

## 5. Experimental Results

This section first provides a description of the environment and libraries used to achieve the experimental results, and then explain how to collect the dataset for evaluation. After that, discusses the evaluation metrics used in the evaluation. Next, discusses the parameter settings used in this paper. After that, compares the topology maps generated via the proposed method with those of other GNG methods in terms of extraction speed and quantization error. Finally, show the results of building a 3D environment map using the proposed method.

### 5.1. Experimental Setup

Open3D [40] was used in this research. Open3D helps display point clouds, and it can perform normal estimation, FPFH feature extraction, RANSAC, and point cloud registration. At the same time, the experiments were run entirely in the Python environment. This experiment was conducted using Ubuntu 22.04 with an Intel Xeon(R) E-2286M processor.

**Figure 12:**
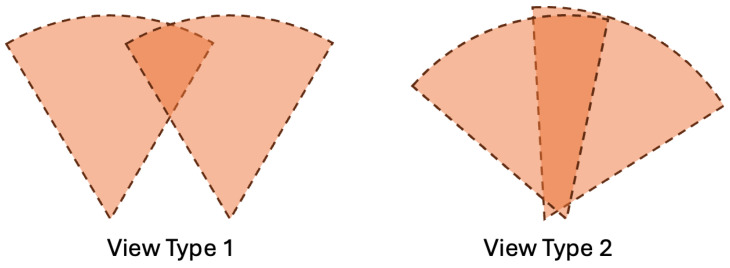
Two different view setups used for the experiments.

Orbbec’s FEMTO MEGA RGB-D cameras were used for this experiment. The colored point clouds were obtained from two view types. In the first view, the two cameras were located at two different positions, observing the same direction with an overlap in the middle. In the other view, the two cameras were set to similar positions but observed different directions. The two view types are shown in Figure 12.

**Figure 13:**
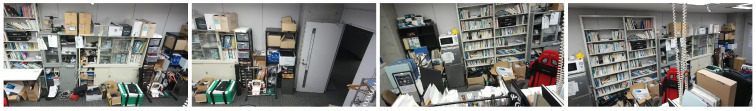
Examples of photos taken from two view types. From left to right, the first two are view type 1, and the second two are view type 2.

**Figure 14:**
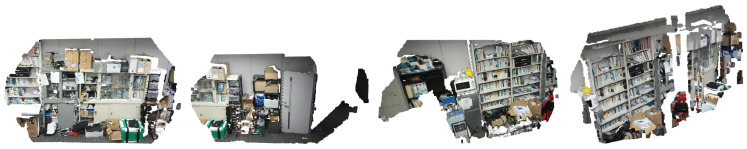
Examples of point clouds taken from two view types. From left to right, the first two are view type 1, and the second two are view type 2.

For each view type, two color point clouds are obtained, and two sample sets are collected for each view type (one sample set contains two point clouds). One of the point cloud in each set is set as the source point cloud and the other as the target point cloud. The goal was to find the best transformation (translate, rotate, and scale) on the source point cloud that matched the target point cloud. An example of captured photos of these two view types is shown in Figure 13. The point clouds of these views are shown in Figure 14. As can be seen from the point cloud example, when multi-camera automatic calibration is processed in a real environment, its point cloud will contain a lot of noisy data, which is suitable for evaluating the proposed method for AAL purposes.

### 5.2. Evaluation Metrics

This subsection discusses how to evaluate the proposed method. To evaluate the performance of fast MS-DBL-GNG compared with other GNG methods, the quantization error [23] and the average computation time (in seconds) were adopted as the evaluation metrics. The quantization error is the average distance between each data (point cloud position) and its nearest node (topological map position), as follows:

(36) $$Err\leftarrow \frac{\sum_{t=1}^{X}\sqrt{{\left(x_{t}-w_{s_{1}}\right)}^{2}}}{\left|X\right|}$$

where X is the position of all points in the point cloud, |X| is the total data size of the point cloud, and $w_{s_{1}}$ is the winner node of the data point. This quantized error is used to measure how well the topological map successfully covers the entire point cloud. Next is the average computation time, which is used to evaluate how quickly the algorithm extracts topological maps from point clouds. This paper present this evaluation metric because fast MS-DBL-GNG can used not only for camera calibration but also for the robotic real-time extraction of topological maps.

Next was evaluating the calibration results. In this study, point clouds were collected by multiple cameras in a real environment, but the ground truth results of the extrinsic camera parameters could not be obtained. Therefore, to obtain the ground truth result, we manually matched the point cloud to the world coordinate system and then saved the transformed point cloud as the ground truth result. The point cloud transformed using the proposed method was compared with the ground truth point cloud, and the average Euclidean distance between each point in the two point clouds represents the error of the proposed method.

### 5.3. Model Parameters

**Figure 15:**
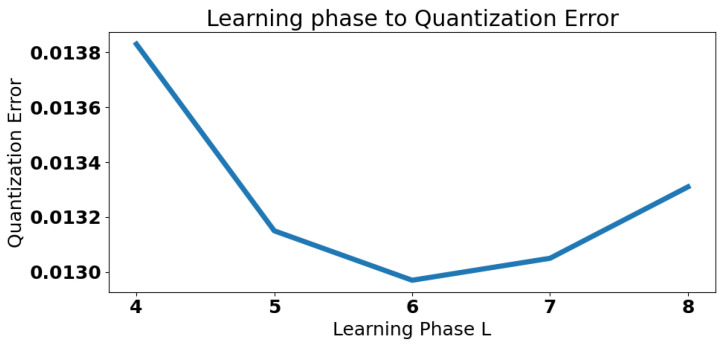
The different learning phase results.

This subsection discusses the parameter settings used in this paper. There are three parameters to consider in fast MS-DBL-GNG. The first is the initial starting point, η. The second is the multi-scale learning phase, *L*. And the last is the maximum number of nodes, *M*. The initial starting point, η, determines how many nodes are pre-placed in the point cloud before learning. When fewer starting points are set, the network will take more time to grow. When too many starting points are set, two starting points may appear in the same area, and it will take time to separate them. Therefore, this paper set the initial starting point, η, to 50. The number of the multi-scale learning phase, *L*, was used to decide how many mini-batches the data were divided into. If it were set too small, the mini-batch size could be too large, and learning would take time; if it were set to be too large, the mini-batch size would be too small, and learning in the first learning stage would be meaningless because, theoretically, a piece of paper can be folded no more than seven times. Therefore, this study set the number of multi-scale learning phases, *L*, to 6. The results of the learning phases are shown in Figure 15. From the figure, it can be seen that learning phase 6 achieved the lowest quantization error compared to the other learning phases.

The maximum number of nodes, *M*, is used to prevent the network from growing too much. If it is set too low, it will cause large quantization errors; that is, the topological map will not be able to cover the entire point cloud. If it is set too large, it will increase the computational cost and the storage capacity. This maximum number of nodes actually depends on the size of the dataset, and it is difficult to determine. Other parameters, such as the learning rate and node error discount rate, had little effect in this study, so this study set the learning rate of the winning node, α, to 0.5 and the learning rate of the neighbor node, β, to 0.1. For the node error discount rates, δ and ρ were set to 0.5.

### 5.4. Topological Feature Extraction Results

This section first evaluates the performance of fast MS-DBL-GNG and compare it with that of standard GNG and other batch learning methods. Two samples from each view type were selected as a data input and downsample them using a voxel grid size of 0.01 before inputting them into GNG. All GNGs require a stopping criterion, so this study set the stopping criterion to be when the number of nodes at the end of the epoch reaches 2000. Each experiment was run five times, and the average results were reported. This study also compared the effects of different learning times on the same mini-batch at each learning phase of the proposed method. The comparison results are shown in Table 4.

From the tabular results, it can be seen that the proposed method, fast MS-DBL-GNG, outperforms other GNG methods. It is at least 17 times faster than standard GNG and at least 96 times faster than other batch learning techniques. This speedup is important because calibration can be done earlier. The quantization error of the proposed method is also lower than that of other methods. A low quantization error means the generated topological map can cover all points in the point cloud on average without losing too much detail. In addition, the tabular results also show that, when fast MS-DBL-GNG learns the same mini-batch twice, the error is smaller than when learning it only once. However, fast MS-DBL-GNG does not show significant results when learning the same mini-batch three times, and the processing time increases. Therefore, this study proposed learning the same mini-batch twice so that GNG could improve its learning ability.

This section also try to compare the results with voxel and Octree algorithms. The difficulty of the comparison lies in the parameter settings of the two algorithms. Voxel requires setting the grid size, while Octree requires setting the depth and expansion size of the tree. Unlike the proposed method, which only need to set the maximum number of nodes to be extracted from the point cloud. Therefore, we tried to set the individual parameters of the voxel and Octree methods based on our approach and finally kept 2000 points (the same as fast MS-DBL-GNG) so that we could compare the results fairly. In this experiment, the entire point cloud was used for fast MS-DBL-GNG, as well as quantization error calculation. Each experiment was run five times, and the average results were reported. The experimental results are shown in Table 5. From the experimental results, it can be seen that fast MS-DBL-GNG outperformed the voxel and Octree methods in terms of the quantization error, but it took a much longer computational time than they did.

**Figure 16:**
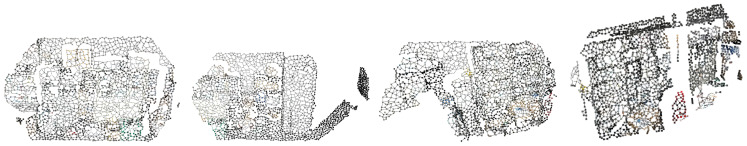
Several examples of topological maps extracted from point clouds using fast MS-DBL-GNG. From left to right, the first two are view type 1, and the second two are view type 2.

The voxel and Octree approaches can downsample point clouds faster than fast MS-DBL-GNG, but the quantization error of fast MS-DBL-GNG is better than theirs. However, the difference is not significant. Nonetheless, a lower quantization error can lead to better calibration results in later stages since calibration is performed on the basis of matched points. If the given point set contains noise and less dense details, multi-camera calibration will fail. In summary, fast MS-DBL-GNG outperforms other GNG methods in terms of quantization error and computation time. However, compared with the voxel and Octree methods, it achieves a better quantization error but a longer computation time. This is a trade-off between time and performance. Figure 16 shows some examples of topological graphs extracted from fast MS-DBL-GNG.

### 5.5. 3D Environmental Map Building Results

This section demonstrates the effectiveness of using topological maps for camera calibration. First, the one-to-one calibration (aligning the source point cloud with the target point cloud) using the proposed method is compared with other methods. Each experiment was performed five times, and the average results were reported. The experimental results are shown in Table 6.

**Figure 17:**
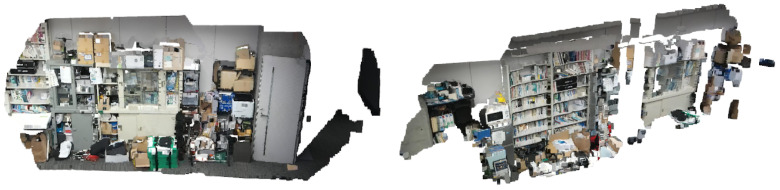
The examples of calibrated point cloud results for view type 1 (**left**) and view type 2 (**right**).

From the results in Table 6, it can be seen that using the topological map achieves smaller distance errors than not using the topological map. As mentioned before, GNG is able to ignore noise and create more nodes in high-density areas, so it can retain important information in the point cloud such that it can be easily calibrated with another topological map. In terms of computation time, it performs faster than other methods because it uses fewer nodes for calibration. Figure 17 shows examples of calibration results for view type 1 and view type 2. From the results of view type 2, it can seen that, although the view is blocked by wires on the ceiling, it is still possible to calibrate to another point cloud.

**Figure 18:**
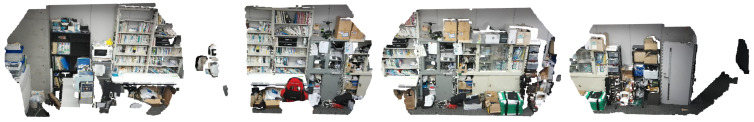
Example point cloud for multi-camera calibration. All of these views are related from left to right or right to left.

This study also proposes a matching strategy for 3D environment map building, which enlarges the original point cloud by merging it with another best-matching point cloud. Therefore, this section also shows the effectiveness of using this strategy. Calibration experiments were conducted using four camera views in view type 1. Two groups of samples were collected, one of which is shown in Figure 18.These point clouds involved a lot of noise, especially in the second photo, where the left side of the view is obscured by wires. The experiment was run five times, and the average results were reported. The experimental results are shown in Table 7.

**Figure 19:**
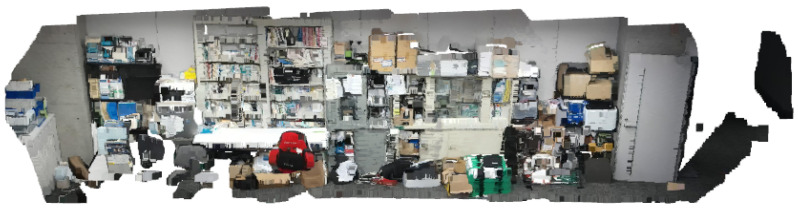
The example of point clouds from four camera views calibrated using the proposed method.

From the results in the table, it can be seen that the error of the proposed method is the lowest among all methods. The lower the error, the better the calibration result, and thus, the method successfully merges multiple point clouds into one coordinate system. From the perspective of calibration speed, the proposed method is slower than fast global registration. The reason for this result is that the proposed method first uses RANSAC and then uses ColorICP for fine-tuning, and RANSAC includes evaluation, while fast global registration does not involve an evaluation at each iteration. Therefore, the proposed method requires more time for calibration in order to obtain better calibration results. However, the proposed method is faster than using the voxel approach. The reason why calibration using the downsampled point cloud of the voxel method is much slower is that the voxel method does not ignore noise and does not build more nodes in high-density areas. Therefore, it takes more time to find and match correspondence points when using the RANSAC algorithm. In addition, after merging with another point cloud, the noise will accumulate, which will increase the RANSAC calculation time again. One of the final results of these mergers is shown in Figure 19.

### 5.6. Discussion

**Figure 20:**
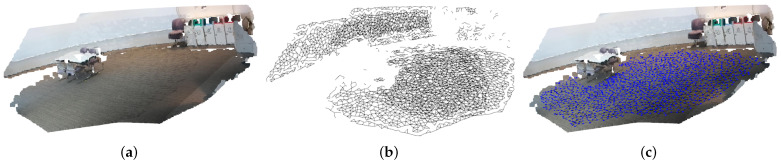
Example of topological map usage for two calibration point clouds. It is easy to distinguish which ones are walkable through the topological map (the blue-colored topological map). From the walkable path, it can be seen that it does not cover the area close to the table, which is an advantage for the robot to navigate. This is a concept of intelligence sensors that provide the required information appropriately to the target. (**a**) Calibrated with two point clouds. (**b**) Merged from two topological maps. (**c**) Extracted walkable area of topological maps.

This section discusses the applicability of using fast MS-DBL-GNG. The main advantage of fast MS-DBL-GNG is that it outputs a topological map that provides edge connections to connect similar nodes together. This feature is not available in the voxel and Octree methods. This topology can be used for clustering, treating unconnected subset nodes as a cluster. Therefore, the extracted topological map can easily distinguish which is a wall and which is a road in the environment. To demonstrate its effectiveness, two cameras were set up in the residential area of a building and then calibrated them into a world coordinate system using the proposed method. Afterwards, based on the extracted topological map, it could distinguish between the walkable path and the wall. This extraction is what we call an intelligence sensor, which provides the appropriate required information for the target. The demonstration examples are shown in Figure 20.

In summary, the topological map can be used to calibrate two point clouds into a world coordinate in a shorter time. In addition, the calibrated topological map can also be used to easily extract walkable paths from the topological map and then share them with robots for navigation tasks. However, the main aim of this paper is to discuss the availability of 3D topological mapping. Therefore, this paper did not evaluate the quality of the obtained topological maps using a mobile robot, which will be a future work derived from this paper.

## 6. Conclusions

This paper has introduced a new method called fast MS-DBL-GNG for a faster topological extraction from point clouds for 3D environment building tasks. First, it performs distributed initialization to place multiple starting points in the point cloud. After that, to reduce data sampling, this study implemented a multi-scale method and learned from each mini-batch to converge the network faster. In batch learning, matrix calculations is applied to speed up the calculation time. At the same time, the idea of different topologies is also implemented, where edge connections and node learning are based on position features. To speed up the growth, multiple nodes were added based on the total number of nodes to be added via the percentile function. Subsequently, in 3D environment construction, first used RANSAC to find the best corresponding points and then used Color-ICP to fine-tune the two topological maps. This paper also introduced a 3D environmental map building strategy to merge multiple camera views into a single world coordinate system by enlarging each topological map and using the best matching topological map.

To verify the performance of fast MS-DBL-GNG, the results of fast MS-DBL-GNG are first compared with those of other GNG methods. The results show that fast MS-DBL-GNG outperforms other methods in terms of quantization error and computation time. Next, fast MS-DBL-GNG is also compared with the voxel and Octree downsampling techniques. From the results, although the proposed method is slower than theirs, but the proposed method obtained a lower quantization error, which is important because it determines the following point cloud calibration results. Then, the calibration of two point clouds into a world coordinate system with and without a topological map are compared. The results show that the proposed method is more stable and takes less time than other methods. This study has also presented a comparison of multi-camera calibration and shown that using topology maps for calibration can reduce the computation time and improve matching accuracy.

There were some limitations in this research. First, this automatic calibration must be performed in an indoor environment with many unique objects; since the proposed method does not use any fixed calibration objects, the calibration process will automatically find the correspondences from the point cloud. Secondly, this study was only tested on Orbbec RGB-D cameras, and the fields of view of multiple cameras must have a certain overlap area. However, there is an advantage of using the proposed method; that is, the topological maps can be shared between robots, for example, in robot navigation. After the multi-camera calibration, the topological maps generated using the proposed method can be used to segment those obstacles and walls from the point cloud, so it can share the traversable path to the robot. Sharing the appropriate information from the sensor to the robot, we can call it an intelligence sensor. In addition, since obstacles can be easily detected through topological maps, they can be used to warn elderly people on a walking path or arrange cleaning robots so that they clear obstacles in order to ensure safety. We will consider these integrations in our future work.

## Figures and Tables

**Table 1:** The main characteristics and differences of several topological mapping methods.

Method	SOM	GCS	GNG	SOINN
Topology Preservation	✓	✓		
Incremental Learning		✓	✓	✓
Topological Clustering			✓	✓

**Table 2:** The main feature differences between variance GNG methods.

Method	Standard GNG	FCM-BL-GNG [21]	MS-BL-GNG [22]	Fast MS-BL-GNG [19]	DBL-GNG [18]
Node initialization	Two random nodes	More than two random nodes	Two random nodes	Three random nodes	**Distributed with more than two nodes**
Node growth frequency	One node per interval	One node per epoch	One node per mini-batch	One node per condition met (iteration)	**Multiple nodes per epoch**
Data sampling	All data per epoch	All data per epoch	All data per scale	**One mini-batch per scale**	All data per epoch
Batch learning strategy	n/a	One by one	One by one	One by one	**Matrix calculation**

The bold text indicates the features to be applied in Fast MS-DBL-GNG proposed in this study.

**Table 3:** Main notations used in fast MS-DBL-GNG.

Notation	Description
*M*	The maximum number of nodes.
*m*	The current number of network nodes.
η	The total number of starting points.
*L*	The total learning phase.
X	All data in the point cloud.
$X^{l}$	A mini-batch of learning phase *l*.
$X^{l,Pos}$	The position features of the mini-batch in the learning stage *l*.
$x_{i}$	Data of index *i*.
*D*	The total number of data.
*W*	Network nodes.
$W^{Pos}$	The position features of network nodes.
$w_{k}$	*k*-th network node.
$E_{k}$	The error of node *k*.
$c_{k,j}$	The connection between node *k* and node *j*.
$\lambda^{l}$	Batch size for learning phase *l*.
ΔW	Accumulate weights to update network nodes.
*A*	The number of activations for the node.
$\hat{c}$	The temporary edge connection.
ϵ	A small positive decimal number.
χ	The node index list.
α	The learning rate of the winner node.
β	The learning rate of the winner node’s neighbors.
δ	The error discount factor.

**Table 4:** The experimental results of different GNG methods.

Methods	Quantization Error	Computational Time
Standard GNG	0.01696 ± 0.00194	40.41041 ± 4.22518
FCM-BL-GNG [21]	0.01767 ± 0.00194	11,662.27850 ± 2798.33940
MS-BL-GNG [22]	0.01742 ± 0.00225	220.92249 ± 8.02279
Fast MS-DBL-GNG^1^	0.02031 ± 0.00326	0.90484 ± 0.28579
Fast MS-DBL-GNG^2^	0.01299 ± 0.00144	2.34518 ± 0.43387
Fast MS-DBL-GNG^3^	0.01264 ± 0.00145	4.13767 ± 0.75200

Fast MS-DBL-GNG* represents the number of times a mini-batch is learned in each learning phase. The proposed method is applied two times.

**Table 5:** Comparison of experimental results between the proposed method and the voxel and Octree methods. The results of fast MS-DBL-GNG were directly extracted from the point cloud without pre-processing through a voxel.

Methods	Quantization Error	Computational Time
Voxel	0.01357 ± 0.00001	0.01872 ± 0.00001
Octree	0.02402 ± 0.00400	0.48581 ± 0.01760
Fast MS-DBL-GNG	0.01145 ± 0.00131	49.88926 ± 0.79331

**Table 6:** One-to-one calibration results (source to target). The calibration results show that using the topological maps extracted via fast MS-DBL-GNG are better and faster than using the voxel method alone and using other methods.

Distance Error			
Method	Fast Global Registration [34]	Voxel	Fast MS-DBL-GNG
View Type 1	0.45546 ± 0.15897	0.33185 ± 0.11623	0.23045 ± 0.12913
View Type 2	0.48632 ± 0.36297	0.43923 ± 0.36283	0.33663 ± 0.24264
**Calibration Time (seconds)**			
**Method**	**Fast Global Registration** [34]	**Voxel**	**Fast MS-DBL-GNG**
View Type 1	1.30145 ± 0.45650	1.56605 ± 0.65369	0.26498 ± 0.34462
View Type 2	1.74838 ± 0.52938	2.03262 ± 0.84651	0.19595 ± 0.11501

**Table 7:** Multi-camera calibration results (four cameras). The results show that multi-camera calibration using the topological map extracted via fast MS-DBL-GNG had the lowest distance error and a faster calibration speed than using the voxel approach alone.

Method	Fast Global Registration [34]	Voxel	Fast MS-DBL-GNG
Distance Error	0.06328 ± 0.03121	0.09531 ± 0.11626	0.02779 ± 0.04742
Computational Time (Seconds)	48.62213 ± 18.27764	1806.24649 ± 576.47094	135.37025 ± 20.09344

## Data Availability

The data that support the findings of this study are openly available on GitHub at https://github.com/CornerSiow/FastMS-DBL-GNG (accessed on 26 August 2024).

## Abbreviations

The following abbreviations are used in this manuscript:

AAL	Ambient assisted living
ICP	Iterative closest point
RANSAC	Random sample consensus
GNG	Growing neural gas
FCM-BL-GNG	Fuzzy-c-means batch Learning GNG
DBL-GNG	Distributed batch learning GNG
MS-BL-GNG	Multi-scale batch learning GNG
Fast MS-DBL-GNG	Fast multi-scale DBL-GNG
